# Effectiveness of harm reduction programmes for injecting drug users in Dhaka city

**DOI:** 10.1186/1477-7517-2-22

**Published:** 2005-10-25

**Authors:** Tasnim Azim, Najmul Hussein, Robert Kelly

**Affiliations:** 1 ICDDR, B: Centre for Health and Population Research, Bangladesh, Mohakhali, Dhaka 1212, Bangladesh; 2 CARE, Bangladesh, Dhaka Field Office, 49/1, Babar Rd, Mohammedpur, Dhaka 1207, Bangladesh; 3 Family Health International, Bangladesh Country Office, House #5, Rd. #5, Gulshan-2, Dhaka 1212, Bangladesh

## Abstract

This paper provides a brief overview of the harm reduction programme for injecting drug users (IDU) of CARE, Bangladesh in Dhaka city and uses data from surveillance and a focussed research study on a cohort of IDU, to evaluate the programme. The harm reduction programme in Dhaka is run by CARE, Bangladesh and includes needle/syringe exchange, awareness raising on HIV/STI, abscess management, condom distribution and advocacy with different groups of people. The needle/syringe exchange programme (NEP) has been in place since 1998, the 2^nd ^Generation Surveillance in Bangladesh is being conducted since 1998, and an in-depth cohort study, started in 2002, is being conducted in two areas of Dhaka city with approximately 500 IDU under CARE's NEP who are being followed bi-annually to assess risk behaviour, incidence of HIV, hepatitis C and syphilis. As the surveillance and the cohort study are both closely associated with the NEP of CARE, Bangladesh, these data can be used to monitor the NEP.

## Review

### Harm Reduction Programme for IDU in Dhaka city

The number of injecting drug users (IDU) in Bangladesh is not known. In a Rapid Situation Assessment conducted in 2002 [1] IDU were found in 20/24 sites studied and although this assessment was not an exercise in enumerating the number of drug users in the country, it was apparent that the maximum number of IDU were in the capital city, Dhaka. CARE, Bangladesh started a needle/syringe exchange programme (NEP) in Dhaka in 1998 covering approximately 880 IDU. This NEP has expanded over the years and in 2004, IDU in 19 districts of Bangladesh were reached by CARE, Bangladesh and at the same time in Dhaka the number of IDU that were being reached had increased to approximately 4400, with 3900 being regular attendees. The NEP services in Dhaka are are coordinated through a central field office and provided through 21 Drop-In Centres (DIC) which are located within or close to communities of IDU and through out reach workers in the field. The services provided include needle/syringe exchange in the field, DIC based clinical services for the management of abscesses and sexually transmitted infections (STIs), drug detoxification camps, condom distribution, education and awareness on the harmful effects of drugs, safe injections, HIV/AIDS, STIs, other blood borne infections. Presently, the average number of needles/syringes given to each IDU in Dhaka is five per week and condoms are being received by 15% of IDU. Of the registered IDU, 55% are married however, there is no specific programme to reach the sex partners of IDU, rather IDU are encouraged to bring their sex partners to the DIC for STI management. In the last year 87 sex partners received STI treatment from the DIC.

In addition to these services, advocacy constitutes a major part of CARE, Bangladesh's intervention programme for IDU. Advocacy is done with members of the community, law enforcing agencies, policy makers from the government, general community leaders, NGOs, and Development Partners as and when required. Special local level advocacy meetings are held at the community level when problems are faced in the field.

In Dhaka, harm reduction services to IDU are provided almost exclusively by CARE, Bangladesh. Other organisations are providing detoxification and some rehabilitation services, and some also provide clinical management for STIs but their clientele are primarily non-injecting drug users.

### Data for evaluating the NEP

#### National Surveillance

Second Generation Surveillance for HIV is being conducted in Bangladesh since 1998 and IDU are one of the population groups sampled in surveillance and they have been accessed from several cities [2-7]. The surveillance system in Bangladesh has the serological and Behavioural Surveillance System (BSS) components, which are conducted in tandem on similar groups, but not the same individuals and the methodologies for sampling for the two systems are different. The serological system sampled IDU exclusively through the NEP of CARE, Bangladesh and most of the IDU sampled in BSS were members of the NEP. Given that the surveillance is so closely linked to the intervention programmes, data from the surveillance system can be used to monitor intervention programmes for IDU, i.e. the NEP of CARE, Bangladesh, as that has the only ongoing harm reduction programme with IDU in Dhaka. Five rounds of surveillance have been completed and as IDU have been sampled from the NEP since the 2^nd ^round (1999–2000), data from the 2^nd ^to the 5^th ^(2003–2004) rounds have been used in this evaluation.

#### Serological Surveillance

The serological surveillance in Bangladesh accesses IDU through intervention programmes [6] and the reason for this is that blood samples cannot be collected in the field setting and results of syphilis tests and treatment for syphilis can only be provided to the IDU in a clinical setting. For serological surveillance, IDU have been accessed annually through harm reduction NGOs with extensive outreach into the community (i.e. CARE, Bangladesh) since the 2^nd ^round (1999–2000) and since the 3^rd ^round (2000–2001) this has been exclusive source of IDU as previous attempts at accessing IDU through detoxification clinics yielded very few IDU.

Serological surveillance has recorded a rise in HIV prevalence over the rounds in IDU from the NEP in Central Bangladesh with prevalence rising from at 1.4% in 1999–2000 (2^nd ^round) to 4.0% in 2002 (4^th ^round) and remaining at 4% during the 5^th ^round (2003–2004) (p < 0.001) [3,8]. Although in the 5^th ^round the HIV prevalence has remained at 4%, in one particular neighbourhood in the same city, the prevalence was recorded at 8.9% (95% CI: 5.4–14.4%) [7]. These findings show that HIV is gradually rising within the IDU in this city and at present most of the cases are localised to one neighbourhood, which may be the epicentre of the epidemic. High rates of hepatitis C have been recorded in the IDU from the very beginning of surveillance and these rates have remained high throughout (66.5% in 2^nd ^round, 62.3% in the 4^th ^round and 59.2% in the 5^th ^round) [7,8]. High hepatitis C rates suggest risky injection and drug sharing behaviours [9]. In contrast to HIV and hepatitis C, active syphilis rates declined significantly over the rounds (9.3% in the 2^nd ^round to1.2% in the 5^th ^round, p < 0.001) [7] suggesting that interventions for sexual risk are possibly being addressed successfully.

#### Behavioural Surveillance System

A two-stage cluster sampling was used in BSS [6]. In the first stage a time location systematic random sampling method was employed to select primary sampling units (PSUs) and in the second stage the numbers of respondents to be interviewed from each PSU was calculated. The PSU for IDU were spots where at least three IDU were found in a specific time frame. Through this method of sampling IDU selected included both those who were in and out of intervention programmes although in the 5^th ^round most IDU sampled were members of the NEP of CARE, Bangladesh. The data used for evaluation are from the 4^th ^and 5^th ^rounds of BSS.

During the 4^th ^round of BSS conducted in 2002, 44.5% (95% CI: 34.6–54.8%) of the IDU sampled from Central city A were under the NEP of CARE, Bangladesh and this increased to 88.3% (95% CI: 84.4–91.4%) during the 5^th ^round in 2003–2004 (p < 0.001). Analyses of data from the 4^th ^round of BSS showed that IDU in interventions practice safer behaviours than those out of intervention [10] and fewer IDU within interventions shared needles/syringes and reported STIs than IDU outside of interventions [11] whereas more IDU within interventions who reported STIs in the last year sought treatment [11]. These findings suggested that the NEP might be having some preventive effect. However, although, coverage increased between the 4^th ^and 5^th ^rounds of surveillance, borrowing of used needles/syringes by IDU during the last week also increased from 65.8% (95% CI: 60.1–71.1%) in 2002 to 86% (95% CI: 82.4–89.0%) in 2004 (p < 0.001). Unfortunately BSS cannot provide reasons for changes in behaviours seen but field observations at the time of surveillance suggested that considerable disruption occurred in the field due to "cleaning" drives by the community and law enforcement agencies which drove the IDU underground from their regular shooting spots. Nine such observations were recorded during mapping and interviewing for BSS over a three month period.

IDU are not only at risk of acquiring HIV through injection sharing but also through unprotected sex [12] which can also allow transmission to other non-IDU partners in their sexual network [13-15]. In Bangladesh, BSS data show that IDU in Central city A are sexually active and in the 5^th ^round of BSS, in the last year 34.5% (95% CI: 29.4–40.0%) bought sex from female sex workers, 2.3% (95% CI: 1.2–4.6%) from male sex workers and 35.7% (95% CI: 29.6–42.3%) had regular non-commercial female sex partners. Although the proportion of IDU who bought sex declined between the 4^th ^and 5^th ^rounds of surveillance (p < 0.001) fewer IDU reported using condoms in the last sex act with female sex workers in the 5^th ^round 15.7% (95% CI: 10.3–23.3%) compared to the 4^th ^round (29.3%, 95% CI: 23.5–35.9%) of surveillance (p = 0.005). These findings indicate that sexual behaviour in those IDU involved in commercial sex has become riskier.

As the BSS sampled IDU from the same areas of the city as the serological surveillance, secondary analyses of the 5^th ^round of BSS data was done to assess whether there were behavioural factors associated with the localised epidemic of HIV recorded by serological surveillance in IDU in a neighbourhood of Central city A (unpublished observations). BSS data from IDU were compared between the neighbourhood with the concentrated HIV epidemic (referred to as area 1) and the rest of the city. In brief, such comparisons revealed that IDU from area 1 were from a lower socio-economic status being significantly less educated, having a lower monthly average income and more commonly living on the street (p ≤ 0.001 for all) than IDU from the rest of the city. In area 1, IDU took more injections on average in the last day and in the last week (p < 0.001 for both) compared to the IDU in the rest of the city although no differences were observed in injection sharing behaviour between the two areas. However, among those IDU who shared in the last week and said they had shared with different partners, the mean number of persons that shared injections were higher in area 1 than in the rest of the city (p = 0.026). Moreover, IDU in area 1 had a lower risk perception than those in the rest of the city. Therefore, there appear to be neighbourhood factors that enhance vulnerability of the IDU to HIV. From these data it is not possible to gage what these factors may be but in other countries factors such as neighbourhood socio-economic status have been shown to play a role in fuelling the HIV epidemic [16,17].

#### Research study on a cohort of IDU

A research study on a cohort of IDU in two areas of Dhaka city within the NEP of CARE, Bangladesh is ongoing. This study has been focussing on the incidence of HIV, hepatitis C and syphilis and risk behaviours of IDU within the NEP. Preliminary findings obtained at the baseline of the cohort study in 2002 showed that there is a localised epidemic (5.9%; 95% CI: 4.2–8.1%) within the cohort of 561 IDU and that in one of the two areas under study the prevalence is 8.0% (95% CI: 5.7–11.1% [18], the same neighbourhood that has recorded an epidemic during the 5^th ^round of surveillance.

Baseline data from the IDU within the cohort showed that 27.7% (95% CI: 24.1–31.5%) and 34.5% (95% CI: 30.7–38.6%) IDU borrowed and lent used needles/syringes, respectively, in the last week despite being in the NEP [18]. Almost half the IDU (48.3%; 95% CI: 44.4–52.6%) obtained their needles/syringes from the NEP as well as drug stores. The reasons for obtaining needle/syringes outside the NEP and for sharing needles/syringes included not having access to outreach workers while injecting (13.2%; 95% CI: 10.0–17.4%), not having needles/syringes with themselves at the time of injection (16%; 95% CI: 12.4–20.4%), not having adequate knowledge about HIV/AIDS (14.2%; 95% CI: 10.8–18.4%), inadequate supply of needle/syringe by the NEP (3.1%; 95% CI: 1.7–5.6%) and a combination of these (33.2%; 95% CI: 28.3–38.5%). In addition, the IDU complained of harassment by law enforcers and the community, poor abscess management, inadequate access to medical treatment and detoxification services. Similar to the BSS data, a significant proportion of IDU in the cohort also reported unsafe sexual behaviour (data not shown).

### Response of CARE, Bangladesh to the data

In response to data from surveillance and the cohort study, CARE, Bangladesh increased its coverage of IDU in Dhaka city and in the neighbourhood where the epidemic was recorded and took some specific actions to improve their programme [19]. To increase the likelihood of having access to outreach workers at the time of injection, the number of outreach workers in the field for distributing needles/syringes were increased from 12 in a month in January 2003 to 100 in December 2003; the number of shifts during which needles/syringes are distributed were increased from one to two daily; and needles/syringes exchange in the field was supervised more closely. To reduce the harassment that IDU face from law enforcing agencies, CARE, Bangladesh increased its advocacy efforts with law enforcers, community and policy makers. Since the study started, monthly visits of IDU in the two study DICs of the IDU increased between January and October 2003 from 337 to 1118 in area A and 548 to 1217 in area B. Similarly, the numbers of IDU whose abscesses were managed increased during the same time at those DICs (from 83 to 278 in area A and 127 to 219 in area B). A hospital willing to provide medical services to IDU such as management of severe abscesses, orthopaedic services, surgery, etc was identified to which IDU are now referred.

Through the cohort study 40 HIV positive IDU have been identified and these HIV positive IDU have raised new demands mainly related to their clinical management and maintenance of confidentiality. Counsellors and a physician from ICDDR,B were appointed especially for their needs and CARE, Bangladesh formed a support group of HIV positive IDU and their partners. CARE, Bangladesh also opened a new DIC for the HIV positive IDU, which is also open for any IDU wishing to attend so as not to discriminate between the HIV positive and negative IDU. Through this DIC medical care and counselling has become much easier and more regular. So far there has been no breach in confidentiality.

## Conclusion

At present the HIV prevalence is still low in IDU but and although in some aspects sex practices may have become safer, injection sharing behaviour has become riskier. Given the high levels of continued risk behaviour and hepatitis C prevalence, the frequent disturbances in the field interfering with the programme activities and that one area of Dhaka city is already experiencing a localised epidemic of HIV, if all out efforts from all quarters are not made, the epidemic is likely to spread in the near future. Available data suggest that the NEP in Dhaka is responsive to the data generated but the responses have been insufficient in changing their injection sharing practices. The harm reduction programme has to develop a more comprehensive response and as there are neighbourhood differences the programme also needs to address a broader environment, which may be making the IDU more vulnerable in that neighbourhood. Consideration of structural interventions may be necessary if an HIV epidemic is to be averted.

## Authors' contributions

TA was responsible for the surveillance and cohort study on IDU and drafted the manuscript.

NH was responsible for managing the NEP of CARE, Bangladesh and was the counterpart for the cohort study on IDU from CARE, Bangladesh.

RK provided technical input to the Behavioural Surveillance and helped draft the manuscript.

All authors read and approved the final manuscript.
